# Microencapsulation of Bacteriophages Using Membrane Emulsification in Different pH-Triggered Controlled Release Formulations for Oral Administration

**DOI:** 10.3390/ph14050424

**Published:** 2021-05-02

**Authors:** Kerry Richards, Danish J. Malik

**Affiliations:** Department of Chemical Engineering, Loughborough University, Loughborough LE11 3TU, UK; k.richards@lboro.ac.uk

**Keywords:** antibiotic resistance, bacteriophages, drug delivery, membrane emulsification, microcapsules, microbiome engineering

## Abstract

An *E.coli*-specific phage was encapsulated in three different pH responsive polymer formulations using the process of membrane emulsification. Small 100 µm capsules were fabricated and shown to afford phages suitable acid protection upon exposure to pH 1.5. Selection of polymer formulations allowed controlled release of phages at pH 5.5, pH 6 and pH 7. Other aspects of phage encapsulation including factors affecting encapsulation yield, release kinetics, acid and storage stability were evaluated. The work presented here would be useful for future evaluation of new therapeutic strategies including microbiome editing approaches allowing pH-triggered release of phages and delivery of encapsulated cargo to different intestinal compartments. The size of the capsules were selected to permit ease of delivery using small bore oral gavage tubes typically used in pre-clinical studies for evaluation of drug substances using small animal vertebrate models such as in mice and rats.

## 1. Introduction

Enteric pathogens including *E. coli*, *S. enterica* and *C. difficile* are common causative agents of food-borne illnesses affecting humans as well as livestock [1]. They flourish in the lower digestive tract and can cause a range of health problems [2]. Although most cases of food poisoning are self-limiting, the risk of developing further complications is greatly intensified due to rising cases of antibiotic resistance in these pathogens [3]. The antibiotic susceptibility of enteric pathogenic bacteria is dramatically declining, necessitating the need to develop and bring to market new therapeutic agents. Pathogenic *E coli* are increasingly causing a range of infections including urinary tract infections (UTI), gastroenteritis and haemolytic uremic syndrome (HUS) [4]. There are 6 known pathotypes of *E. coli*, all distinguished by their mechanism of infection including for example, toxin production, adherence and invasion strategies. All pathotypes are becoming progressively resistant to frontline antibiotics and are increasingly likely to become untreatable in the future if new alternative treatments are not forthcoming [2]. Diarrhoea already causes devastating mortality rates in low-income countries and is also of increasing concern in higher-income settings. This is a result of increased opportunity for travel, resulting in worldwide spread of pathogenic strains [5]. The decreasing sensitivity of bacteria to antibiotics and increasing risk of difficult to treat infections will intensify in future, the need for new treatments for pathogenic *E. coli*.

Looking from a different perspective, the human microbiome has been reported as an essential aspect of physiological health, with microbiome disruption making way for opportunistic infections. Microbiome engineering refers to the alteration of microbial communities, through supplemented or subtractive approaches, to provide an overall improvement to human health and agricultural productivity [6]. Targeted removal of pathogenic bacterial strains without impacting the diversity of the microbial community would provide a novel infection treatment approach, whilst reducing the incidences of subsequent opportunistic infections. This is specifically applicable to *E. coli*, which has a synergistic interaction with its host, yet occurrence of pathogenic *E. coli* strains causes severe health complications [7]. Removal of a specific pathogenic *E. coli* strain, without disruption to the microflora diversity would be an attractive therapeutic approach currently not achievable using broad-spectrum antibiotic therapy.

Bacteriophages (phages) are viruses that are specifically adapted for infecting bacterial cells. Lytic phages infect highly specific strains of bacteria hijacking bacterial replication machinery to produce progeny phages. Phage encoded enzymes produced during phage replication direct bacterial cell lysis thereby releasing progeny phages into the environment [8]. The amplified phages can continue to infect the remainder of the bacterial population [9]. The therapeutic potential of bacteriophages as antibacterial agents has previously been shown for a range of infections for both Gram-positive and Gram-negative bacteria [10,11,12]. Phage cocktails (employing a number of different phages) are typically used for reduction of bacterial loads where different bacterial strains may be present thereby providing a broader host range coverage [13,14,15,16]. However, a limiting factor in the development of effective phage therapeutics continues to be effective phage delivery systems whereby high concentrations of active phages are delivered directly to the site of infection. Phages are susceptible to environmental stresses encountered in vivo including exposure to gastric acidity, degradative enzymes and dilution of the dose en route to the site of infection [17].

Previous encapsulation efforts have demonstrated significant improvements in phage protection from acid exposure. However, limited work has heretofore been published showing how through careful selection of encapsulation material, controlled release of phages may be achieved triggered by differences in pH in the different gut compartments, e.g., small intestine ((proximal pH 5.8–6.5); distal (pH 6.6–7.5)) or in the large intestine (pH 6.4–7.0) [17]. Microencapsulation refers to the packaging of materials in micrometer-sized capsules with encapsulation material chosen such that release of encapsulated cargo can be achieved under specific conditions. Microencapsulation of *S. typhimurium* phage Felix O1 in a chitosan-alginate formulation was previously shown to improve phage activity upon exposure to simulated gastric fluid (SGF) and bile salts in an in vitro study [18]. The formulation was not designed for targeted release. Spray drying has been used to produce dry powder oral dosage forms for enteric delivery [19]. Phage Felix O1 was spray dried in a pH-responsive polymer and the spray dried powders were tableted using a direct compression process. However, up to 99% reduction in phage titre (2 log loss) was observed in the spray dried powders after their exposure to SGF; this loss may be attributable to thermal and drying stresses during the spray drying process. These issues highlight the need for further development of better formulations and encapsulation methods for bacteriophages for targeted delivery applications.

The potential of phage encapsulation using scalable membrane emulsification technology has only recently been investigated [19]. Phages were successfully encapsulated in pH-responsive microcapsules produced using membrane emulsification employing a commercially available Eudragit polymer S100. The study demonstrated phage survival after exposure of the microcapsules to SGF. The polymethylmethacrylate co-methacrylic acid polymer formulation used in the study would only permit phage release at pH greater than pH 7 thereby targeting phage delivery to lower parts of the GI tract. Food grade pH-responsive polymers are widely available and could be selected based on different chemistries that may allow phage delivery to different parts of the GI tract including delivery to earlier sections of the human gut, e.g., release in the small intestine [20]. To date, such formulations have not been evaluated systematically for phage encapsulation and controlled release applications.

The aim of the present study was to demonstrate encapsulation of an *E. coli* phage T3 belonging to the *Podoviridae* family in different pH responsive formulations using a membrane emulsification process. Three different polymer formulations from the Eudragit series were utilized; these may allow delivery of phages targeting different compartments of the human gut based upon a pH-triggered burst-release mechanism. Successful encapsulation in three different polymer formulations would enable high doses of phages to be delivered to specific compartments of the GI tract including the duodenum (L100-55), jejunum (L100), ileum and colon (S100). Microcapsules may also allow pre-clinical evaluation of therapeutic strategies in small vertebrate animal models such as mice and rats where capsules need to be small enough to allow oral dosing using small-bore oral gavage tubes. The suitability of the membrane emulsification process to produce uniform microcapsules using the different polymer chemistries was investigated. In vitro evaluation of pH dependent phage release was investigated. The phages encapsulated in the microcapsules were tested for their protection against gastric acidity and their stability during storage.

## 2. Results

### 2.1. Production of Similar Sized Microcapsules with Different Polymer Formulations

**Figure 1:**
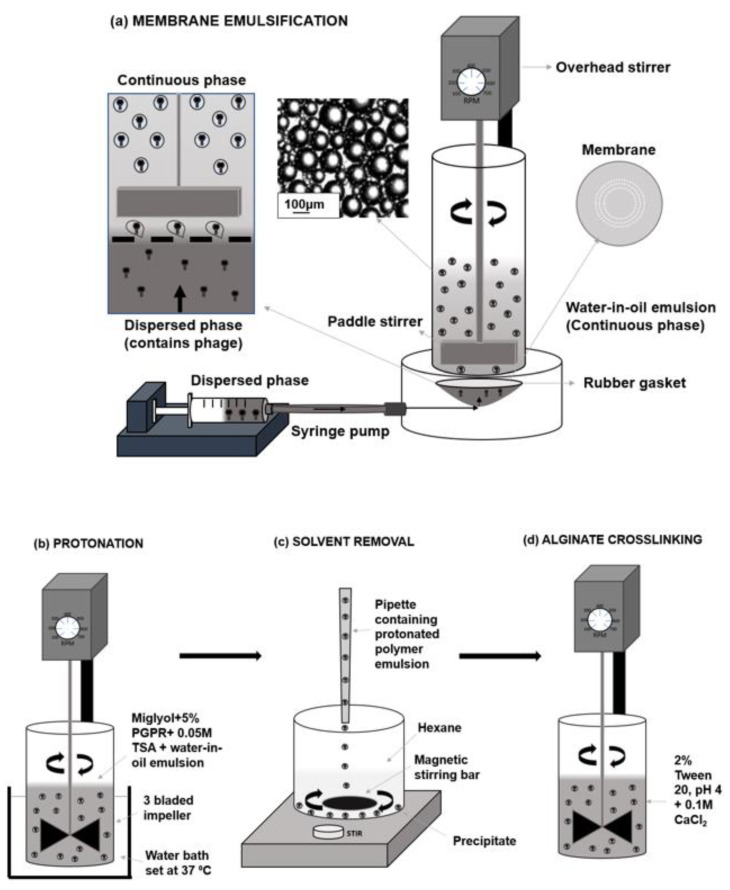
Schematic of the capsule production method. (**a**) The membrane emulsification system—a dispersed phase containing the polymer formulated phage suspension was pumped upwards through membrane pores into a continuous (oil) phase. A paddle stirrer created shear at the membrane surface, forcing droplet detachment. The continuous phase contained PGPR as emulsifier to stabilise droplets after detachment. (**b**) Polymer precipitation was induced by protonation using TSA—the emulsion was added to a vessel containing the acidified oil to enable capsules to form as all the polymers protonate upon acidification resulting in production of microcapsules. A water bath ensured the protonation reaction temperature was controlled. A 3-bladed impeller was utilised for axial mixing. (**c**) Solvent removal—TSA protonated capsule suspensions were slowly added into a vessel containing hexane to allow removal of miglyol. A magnetic stirring bar gently agitated the capsules as they collected at the base of the vessel to reduce capsule agglomeration. Hexane/miglyol mixture was subsequently removed and the capsules were recovered. (**d**) Alginate crosslinking—hexane washed capsules were resuspended in a 2% (v/v) Tween-20 solution (pH adjusted at pH 4) with the addition of CaCl_2_ (0.1 M final concentration). The alginate in the capsules hardened, forming an insoluble hydrogel matrix resulting in the final microcapsules.

**Figure 2:**
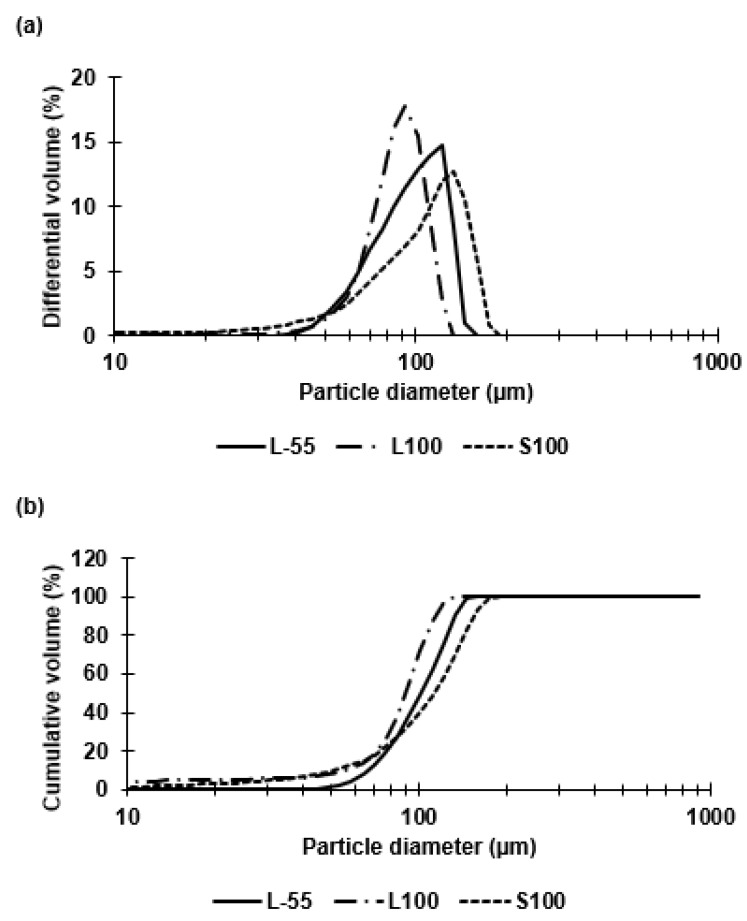
Particle size distributions of droplet diameters for each emulsion, measured using the Coulter LS series. Emulsions produced from Eudragit polymers: L-55, L100 and S100 using a paddle rotation speed of 250 rpm, and a dispersed phase flow rate of 25 mL h^−1^. The dispersed phase composition was 10% (*w/v*) Eudragit polymer and 1% (*w/v*) medium viscosity alginate. The continuous phase composition was Miglyol: castor oil ratio 9:1 with 5 v/v % PGPR. (**a**) differential volume, (**b**) cumulative volume.

Three different synthetic polymers with different pH dissolution profiles were evaluated in the present study, thereby allowing stratified pH-dependent phage release dynamics. Measurement and analysis of the capsule size distributions was carried out for each polymer water-in-oil microemulsion and the resulting capsules that were produced (Figure 1). The goal was to ensure similar sized microcapsules (mean size ~ 100 µm) were used throughout the study in order to provide a like-for-like comparison in terms of acid stability and phage release kinetics from the capsules prepared using the three different polymers. The mean size and size distributions of the primary water-in-oil emulsions produced using a 40 µm pore membrane were similar for the three different polymer formulations (Figure 2).

The membrane emulsification conditions were kept constant for each polymer, with a set dispersed phase flow rate of 25 mL/h and a stirrer speed of 250 RPM. The volumes of dispersed phase and continuous phase used were consistent throughout. The shear force at the membrane surface affected the aqueous droplet detachment at the membrane surface and this was controlled by the paddle rotation rate and solution viscosity (mainly influenced by the alginate composition which was the same in the three formulations at 1% (*w*/*v*)). All polymers tested produced similarly sized droplets with mean values within ±15 µm of 100 µm and CV values between 30–40% (Table 1). The span values for L-55 and L100 droplets were similar at 0.64 and 0.62, respectively, however, the span value for S100 droplets was slightly higher ~1.0. Microcapsules around 100 µm in size can easily be administered through a typical 18 gage oral gavage needles whereas larger microcapsules cannot and therefore these capsules would be suitable for experimentation in small animals such as mice used for preclinical evaluation of phage biotherapeutics.

### 2.2. Effect of Protonation Conditions on Acid Stability of Phages in Microcapsules

After production of the water-in-oil emulsions, they were added to a solution of acidified oil to protonate the different Eudragit polymers (Figure 1a,b). The addition of alginate to the formulation offered acid protection for the phages during this process; in the absence of alginate in the formulation, viable phages were not recovered from the capsules (Supplementary Materials, Figure S1). The temperature and time for protonation were factors that were varied during initial optimisation of protonation conditions to investigate their effect in terms of affording phages protection from gastric acidity. Microcapsules were protonated for 2, 4 and 6 h at two curing temperatures (25 °C and 37 °C). Capsules produced at 25 °C were found not to provide adequate acid protection to phages after protonation for 2 h and 4 h (Supplementary Materials, Table S1). Protonation for 6 h at 25 °C did improve phage survival upon exposure to SGF, however the length of treatment in the acidified oil reduced the phage titres recovered after the protonation step (Supplementary Materials, Table S1). Protonation of capsules at 37 °C was considerably faster and resulted in solid capsules after 2 h of protonation and conferred stability to the encapsulated phages upon exposure to SGF. Increasing the protonation time at 37 °C to 4 h or 6 h resulted in a significant loss of phage titre due to the protonation step with no significant improvement in acid stability. 2 h of protonation at 37 °C in acidified oil was found to be the best set of conditions for the protonation step and was therefore used to produce the capsules for the results presented in this study.

CaCl_2_ crosslinking of alginate was carried out for 1 h after the protonation step at a final CaCl_2_ concentration of 0.1 M. Increasing crosslinking time to 4 h did not offer any improvements in terms of acid stability but was found to result in greater agglomeration of the capsules (data not shown). Hence, capsules produced in this study were crosslinked using 0.1 M CaCl_2_ for 1 h. These production conditions were found to be optimal across all three formulations and provided high phage yields in the capsules and stability during the production process, as well during further testing with SGF. During handling and storage, the capsules displayed a tendency to agglomerate after the alginate gelation step even though capsules were thoroughly washed with aqueous acidified tween 20 (pH 4) solution following gelation to remove excess calcium chloride. S100 and L100 formulations were considerably more uniformly dispersed (SEM analysis, Supplementary Materials, Figure S2). L-55 capsules tended to aggregate significantly following the calcium chloride gelation step.

### 2.3. In Vitro Testing of Encapsulated Phage Release under Simulated Gastrointestinal Conditions

**Figure 3:**
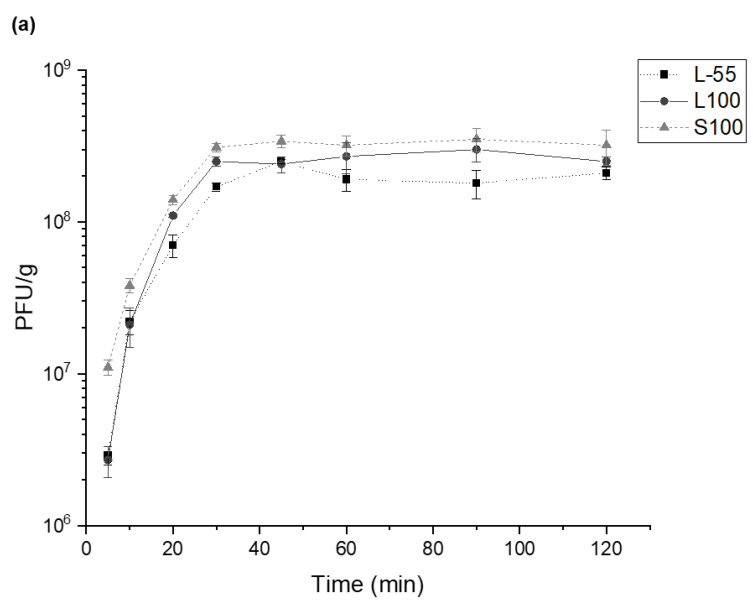

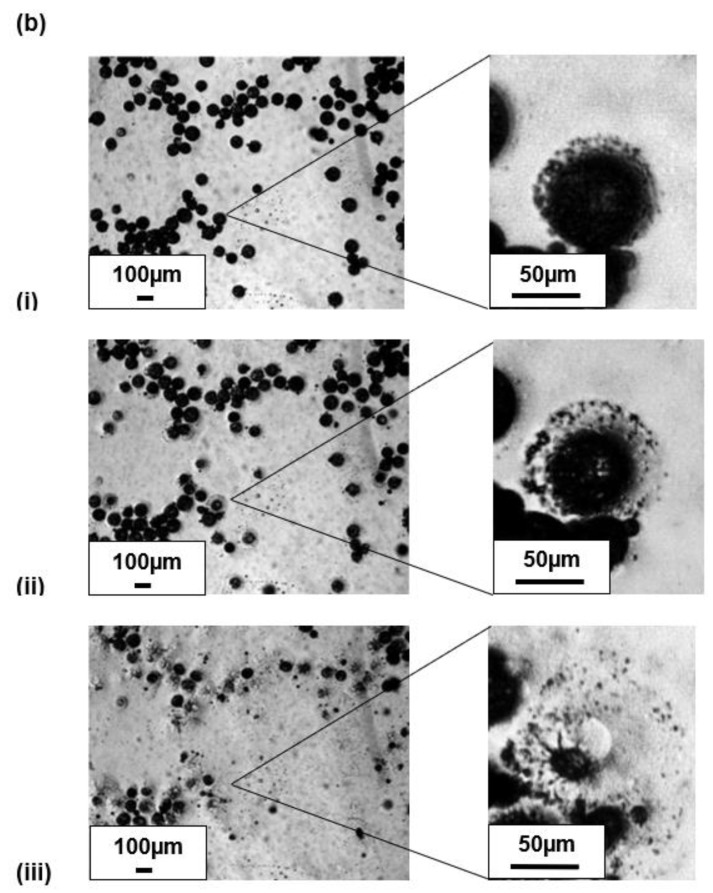

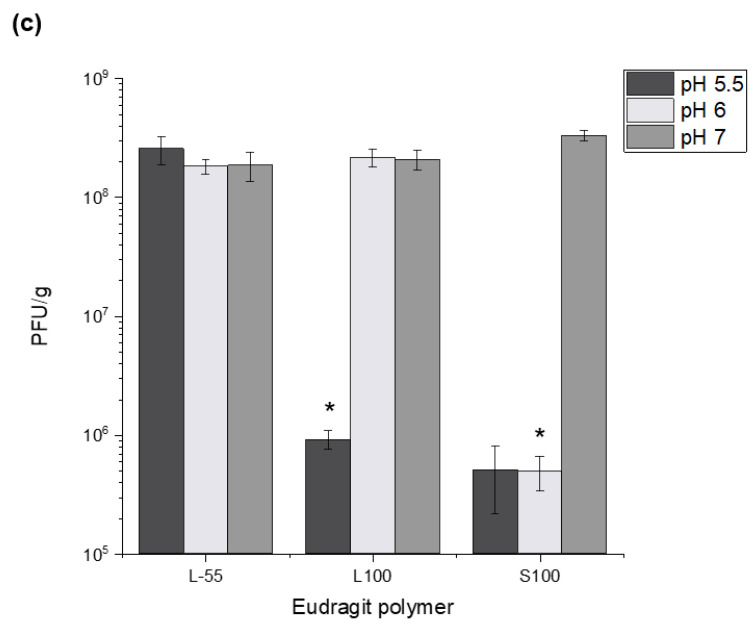
In vitro phage release data from the three different polymer formulations. (**a**) Release kinetics of encapsulated phage in polymers L-55, L100 and S100. T3 containing capsules were exposed to simulated intestinal fluid formulated mimicking different gut compartment pH values (L-55: pH 5.5, L100: pH6, S100: pH 7) for 2 h and the released phage titres determined over time using plaque assay method. (**b**) Microscope images were captured using a ×10 magnification lens and a Phantom C100 high speed camera. S100 capsules were suspended in 2% (*v/v*) Tween 20 (pH 4) before Sorensen’s buffer (pH 7.5) was added to the sample in situ. Images were captured at 0 s (**i**), 20 s (**ii**) and 40 s (**iii**) to observe capsule dissolution kinetics. (**c**) The final amount of phage T3 per gram of capsules released upon exposure to solutions with different pH. 0.1 g of capsules were suspended in 1ml SIF and final concentration of phages released was measured after 2 h incubation upon dissolution of capsules. Error bars represent one standard deviation. * Significantly different phage titres using a paired *t-test*.

Encapsulated phages in each of the three formulations were exposed to solutions mimicking different simulated intestinal compartment pH conditions. Complete release of phages from the different polymer capsules was achieved within 30–45 min of exposure of the capsules to different simulated intestinal compartment pH values, (pH 5.5 (L-55), pH 6 (L100) and pH 7 (S100)) (Figure 3a)). The amount of phages (dosage) in the capsules were found to be similar with encapsulation of approximately 10^8^ PFU/g of T3 phages in capsules made using each of the formulations. This equated to around 50% encapsulation yield compared to the theoretical amount achievable, based on phage addition to each formulation prior to capsule production; thus, significant phage loss was experienced during the capsule production process.

High speed imaging provided real-time observation of the capsule dissolution process. Representative time series set of images were taken for S100 capsule dissolution upon exposure to pH 7.5 Sorensen’s buffer (Figure 3b images i–iii). Capsule dissolution was clearly observed over a time scale of 0–40 s following addition of the buffer suggesting a rapid burst release at this pH. Agglomerated capsules showed slower rates of dissolution (data not shown) suggesting that the local pH in the environment of the capsules may be different to the buffer and may influence the rate of dissolution for the agglomerated capsules.

The pH dependent release of phages attributed to the different formulations was confirmed (Figure 3c). L-55 capsules released encapsulated phages at pH 5.5 and above whereas significant release of phages for the L100 formulation was noted at pH 6 and above and for S100, pH 7 and above (Figure 3c). These results demonstrate the potential of the different formulations to provide targeted delivery and controlled release of encapsulated phages provided there is a clear stratification of pH in the GI tract, e.g., differences in pH between the small and large intestinal compartments. 

### 2.4. In Vitro Testing of Encapsulated Phage Survival upon Exposure to Simulated Gastric Fluid and Phage Release Dynamics Thereafter

**Figure 4:**
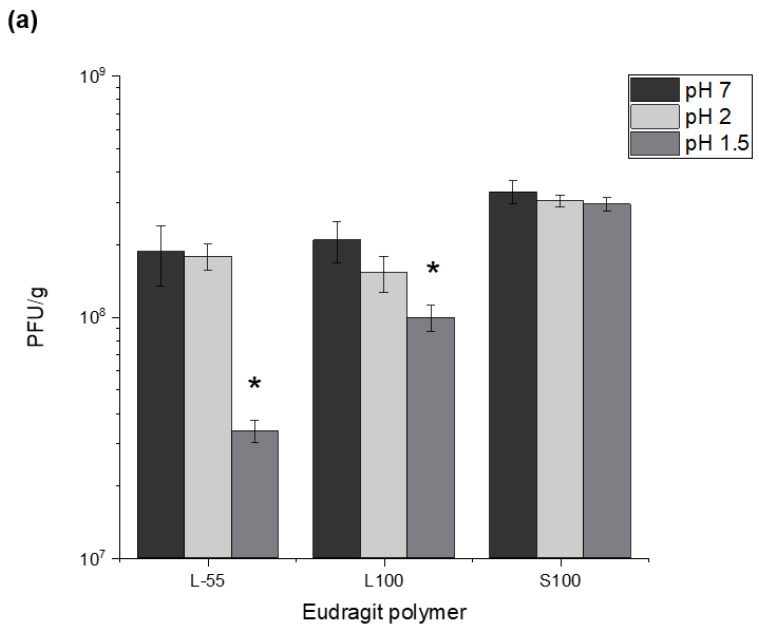

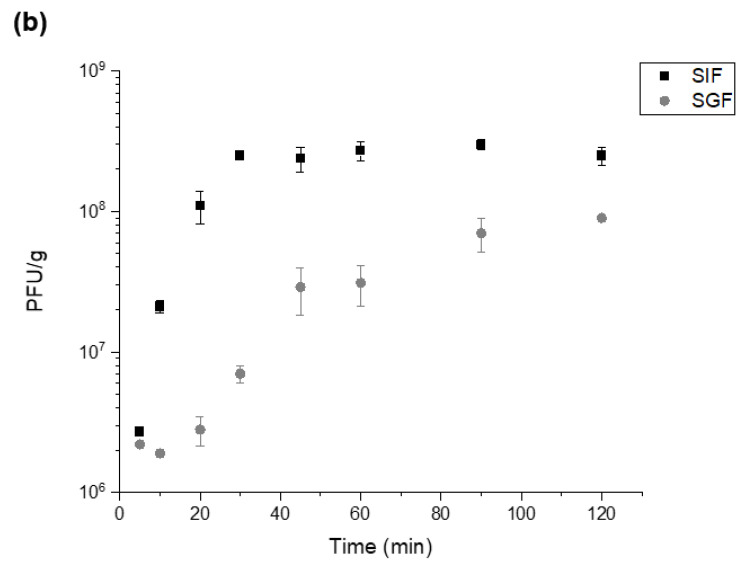
In vitro measurements of acid protection and encapsulated T3 phage release kinetics from microcapsules. (**a**) concentration of viable T3 phages released from capsules before and after exposure to SGF (pH 1.5 or pH 2). Capsules were exposed to SGF for 2 h. Subsequently, capsules were exposed to SIF (pH 7) and the amount of viable phages released were measured after 2 h at which point the capsules had dissolved completely. (**b**) Release kinetics of encapsulated phage T3 released from L100 capsules after SGF exposure. Capsules were exposed to SGF (pH 1.5) for 2 h, SGF was subsequently removed. Capsules were then resuspended in SIF and phage concentration measurements were taken over a duration of 2 h. Error bars represent one standard deviation. * Significantly different phage titres using a paired *t-test*.

Successful enteric delivery of encapsulated phages relies on the passage of the capsules through the stomach en route to the low GI tract. Orally administered phages are subjected to a harsh gastric environment due to digestive enzymes and the low stomach pH therein. Capsules containing encapsulated phages were incubated in simulated gastric fluid (SGF) for 2 h (at two different pH conditions, pH 1.5 and pH 2) to determine the viability of phages in the capsules after gastric exposure. Differences in recovery of T3 phages following acid exposure of the capsules was compared with capsules not exposed to an acid environment (Figure 4a). There were significant losses in phage activity in capsules formed using formulations L-55 and L100 compared with S100. All capsules provided the phage protection against acid exposure at the low acidic pH 1.5 for 2 h (pH and residence time typical of stomach conditions in humans). A significant difference was observed between the initial phage titres and the phage titres after incubation with pH 1.5 buffer for formulations L-55 and L100. S100 exhibited minimal phage loss at both pH 2 and 1.5, with no statistically significant difference in measured phage titres with and without SGF exposure. L-55 displayed a 1 log reduction at pH 1.5, compared with a 0.5 log reduction with L100. Exposure to pH 2 caused minimal loss in phage activity for both L-55 and L100 (no statistically significant difference). Thus, for all 3 polymers, complete phage survival was demonstrated at pH 2 exposure however, there were significant phage losses due to exposure at pH 1.5 for L-55 and L100.

The capsules appeared considerably more agglomerated following exposure to gastric fluid (visual observation under an optical microscope, images not shown). Release kinetics from SGF (pH 1.5) were measured for L100 capsules (Figure 4b) and the rate of release compared with capsules not exposed to SGF. The kinetics of phage release from the L100 capsules after SGF exposure was found to be significantly slower compared with capsules that had not been exposed to SGF. Complete release of phages from SGF exposed capsules took approximately 90 min in Sorensen’s buffer (pH 7).

### 2.5. Phage Activity and Aggregation of S100 Capsules Stored for 6 Months in the Fridge

Phage viability in capsules during storage is an important consideration. Weekly tests followed the phage viability over time to determine whether the capsules provided a sustainable environment for phage survival during storage. Initially capsules were refrigerated in pH 2.5 buffer with 2% tween 20 but T3 was undetectable after 1 week of storage, hence pH of the storage buffer was increased for the study going forward. Storage in 2% tween 20 at pH 3.5 enabled survival for 4 weeks of storage, however a 1 log 10 decrease occurred by the 4th week. One concern was the occurrence of phage release if the storage pH was increased too much, with the pH elevating over time of storage. Initial phage storage solutions were at pH 3.5, yet after the 4 weeks of storage the buffer pH had risen to pH 3.9. Testing phage release when exposing the capsules to pH 3.5 Sorensen’s buffer concluded that no phages were detected, thus storage in pH 3.5 would not result in polymer dissolution.

**Figure 5:**
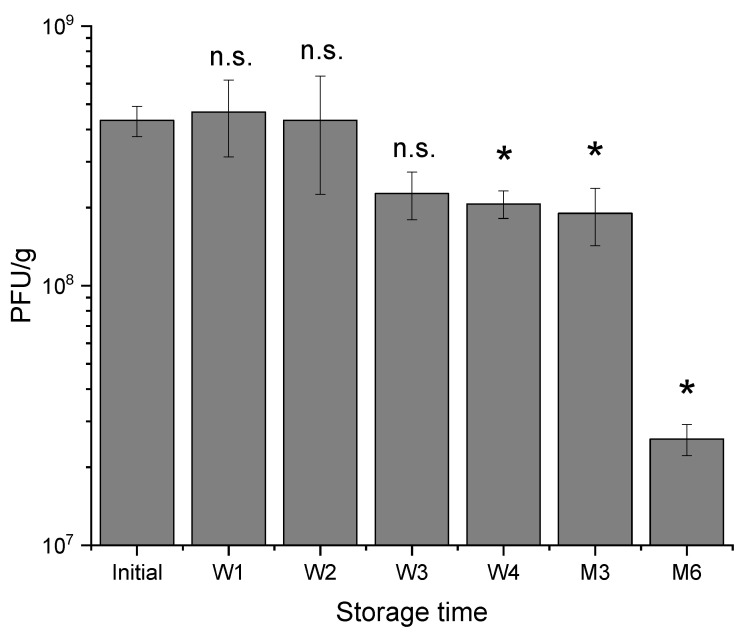
Phage activity in S100 capsules stored for 6 months at 4–8 °C in the fridge. Capsules were stored after wicking any residual moisture using filter paper. Weekly measurements (measured for the first month) of T3 phage release following dissolution of capsules for 2h in Sorensen’s buffer (pH 7). Measurements were subsequently taken at months 3 and 6. Error bars represent one standard deviation. N.B. No significant difference between phage titres compared to the initial titre using a paired *t-test*. * Significant values (*n* = 3), n.s. not significant.

To improve the storage stability further, capsules were stored without any tween 20 storage liquid. After production, the capsules in 2% tween 20 were transferred to falcon tubes and placed upright. The capsules settled to the base of the tube and the tween 20 solution was carefully aspirated out using a pipette. As much of the liquid was removed as possible and the hydrogels were refrigerated for 4 weeks. This method improved the storage stability of the capsules with minimal losses being experienced such that the phage titre decreased from an initial 4 × 10^8^ PFU/g to 2 × 10^8^ PFU/g in week 4 (Figure 5). A slight decrease was observed after 3 months of storage, however a 1 log decrease was measured after 6 months of storage.

### 2.6. Capsule Agglomeration under Refrigerated Storage Conditions

**Figure 6:**
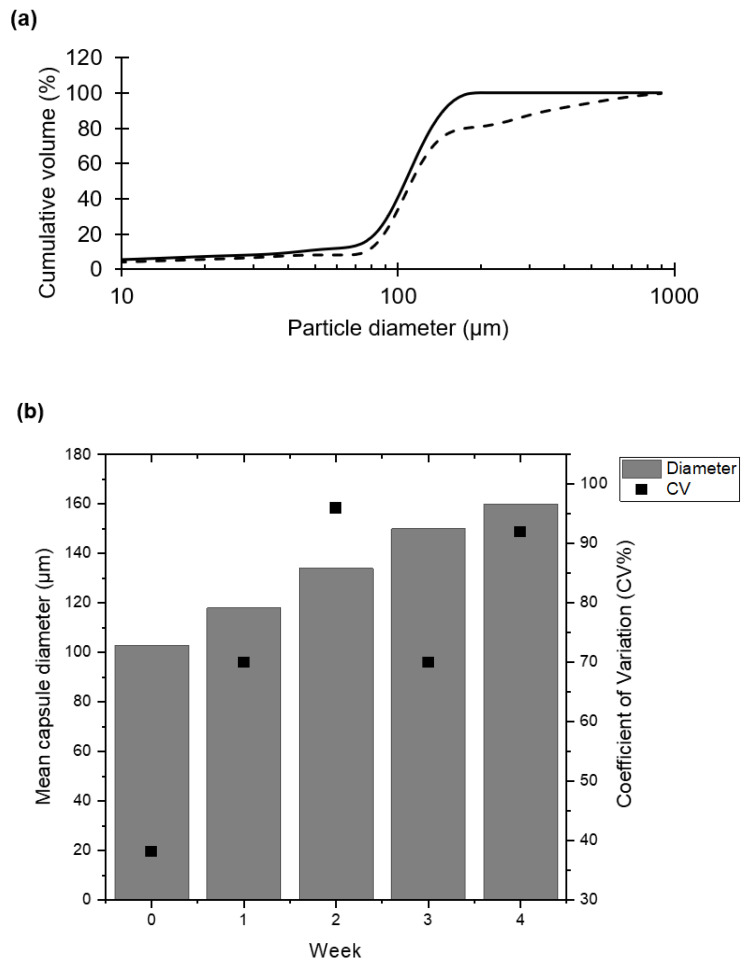
Evaluation of capsule agglomeration during storage under refrigerated conditions (4–8 °C). Size distributions of capsules containing phage T3. Size distribution of capsules were measured immediately after production. Capsules were stored dry (any free liquid removed by pipette). Particle size analysis was carried out weekly for 4 weeks. (**a**) The particle size analysis is presented as a cumulative volume distribution. The solid line represents data for capsules immediately after production. Dashed line represents data for capsules stored for 4 weeks. (**b**) Mean particle sizes displayed as bars, ‘solid squares’ represent the coefficient of variation (%).

Agglomeration of capsules during storage causes difficulties in oral administration of the capsules in small animal models using a gavage tube. Initial visual observations suggested differences in agglomeration rates during storage at room temperature compared with refrigerated conditions. The room temperature stored samples showed a greater tendency to agglomerate, with aggregates too large to pass through an 18 gauge oral gavage tube. Consequently, capsules were stored under refrigerated conditions only. The extent of aggregation was monitored each week over a period of four weeks (Figure 6a). The original capsules had a mean diameter of approximately 100 µm and showed a narrow size distribution and a low CV value. After 4 weeks of refrigeration, the recorded cumulative distribution shifted to the right indicating an increase in the capsule sizes due to the agglomeration of individual capsules (Figure 6a).

Changes in mean capsule diameter and corresponding CV values were recorded over the 4-week storage period (Figure 6b). The mean capsule diameter increased over time from a starting value of ~100 µm to ~160 µm by week 4. The corresponding CV values also increased from ~40% to ~90% indicating greater capsule size heterogeneity due to capsule agglomeration. The rate of agglomeration could be slowed through washing of capsules with 2% (*v/v*) tween 20 in order to remove residual CaCl_2_ and using refrigerated storage (data not shown).

## 3. Discussion

Microencapsulation techniques suitable for phage encapsulation, targeted delivery and controlled release are essential for future phage therapy. Future developments utilising phages for targeted gut microbiome engineering offer exciting therapeutic opportunities. Capsules encapsulating phages and displaying pH-triggered burst release dynamics may prove particularly beneficial in delivering high payloads of phages in a targeted manner. The phage therapeutic cargo needs protection from environmental stresses such as the stomach acidic environment and exposure to digestive enzymes *en route* to the delivery site downstream in the small or large intestine. Biopolymers such as alginate have previously been used in pharmaceutical preparations due to their ease of biodegradability, biocompatibility and their ability to act as a carrier for the therapeutic agents [21]. Commercially available Eudragit polymers L-55, L100 and S100 have been specifically designed for enteric delivery applications with the aim of protecting therapeutics from gastric acidity and allowing controlled release of therapeutics utilising a pH-dependent trigger mechanism. In the present study, alginate and Eudragit polymers were used in combination to produce pH-responsive microcapsules which were shown to protect phages upon exposure to simulated gastric acidity whilst releasing the phage payload when the environmental pH was increased.

Capsules of similar size (~100 µm) were prepared for each of the three polymer types (L-55, L100, S100). The polymer type had no significant effect on the resulting capsule size during emulsion generation using membrane emulsification. The W/O emulsion droplet size and size distribution dictated the final size of the capsules. Size distribution analysis confirmed size heterogeneity due to the presence of small droplets formed during the emulsion production process and a non-gaussian shape of the size distribution curve. The majority of the droplets however, were approximately 100 µm in diameter. The membrane emulsification system used here results in generation of primary droplets at the membrane surface that are membrane pore size dependent, however, secondary droplet break-up in the batch emulsification vessel may result in formation of smaller droplets due to interaction with the impeller following primary droplet formation and seen as a tail in the volume size distribution thereby resulting in the measured non-gaussian shape of the size distribution curve. An objective of this study was to compare the performance of similar sized capsules across the three polymer types to access the degree of acid protection and targeted enteric delivery based upon pH triggered release. The three polymers differed in composition, with L-55 based on a copolymer of methacrylic acid and ethyl acrylate whereas L100 and S100 were copolymers of methacrylic acid and methyl methacrylate with different amounts of carboxylic acid residues providing differences in pH dissolution characteristics. The ratio of free carboxyl groups to ester groups is different for these polymers (L100 1:1, S100 1:2) [22,23]. The differing compositions resulted in release of phages at different pH values of pH 5.5 (L-55), ~ pH 6 (L100) and ~ pH 7 (S100). Three Eudragit polymers were selected to enable targeted release in different locations of the GI tract. L-55 dissolution occurs above pH 5.5 thereby allowing duodenum targeting of the drug substance, L100 microcapsules should release cargo in the jejunum and S100 in the ileum and colon [24]. The pH responsive characteristics of Eudragit polymers depend upon the pKa of the carboxyl group, enabling dissolution at different pH values. As the pH of the local environment changes in different sections of the GI tract, different formulations of Eudragit polymer capsules would dissolve in a particular pH range thereby enabling burst release of the encapsulated phage cargo at specific different locations allowing targeted delivery (Supplementary Materials, Figure S4).

Previous studies using membrane emulsification have reported CV values of around 10–30% (for O/W emulsions) [25] though the components of the dispersed and continuous phases were different to this study. The alginate added to the dispersed phase was found to be essential for phage acid protection but resulted in an increase in the dispersed phase viscosity impacting on the uniformity of the generated droplets [26]. The formulation was specifically designed for bacteriophage encapsulation with 1% (*w/v*) alginate offering good protection during TSA protonation, whilst facilitating a droplet size of approximately 100 µm. A previous study compared acid protection of encapsulated phages in alginate-S100 (using 1% (*w/v*) alginate) capsules with two different sizes (100 µm and 50 µm) and concluded that the larger capsules offered increased acid stability [27]. Thus, size of capsules is a critical factor affecting phage viability during gastric transit. The present study builds on this earlier work extended previous work through comparing three different polymers and using a different enteric *E. coli* specific phage from the *podoviridae* family. The addition of 10% (*v/v*) castor oil to the continuous phase increased the dispersed phase viscosity (19 cP), increasing the shear stress at the membrane surface thereby allowing greater control over production of 100 µm sized droplets using a 40 µm membrane. A ringed pore membrane allowed more precise control over the surface shear in the vicinity of droplet generation site resulting in lower CV values for emulsions compared with membranes where the holes were evenly distributed over the entire membrane surface area.

Eudragit polymers have been used extensively within the pharmaceutical industry for gastrointestinal delivery of therapeutic agents, however, their use in phage encapsulation has only recently been investigated. Targeted delivery of enteric phages at specific locations of the GI tract would be beneficial when treating enteric infections which may reside specifically in different parts of the gut. Treatment of bacterial infections and bacteria associated gut dysbiosis using more specific narrow spectrum targeted approaches is preferred to using broad spectrum antibiotics which do not discriminate between pathogenic and beneficial bacteria. Cocktails of therapeutic phages are increasingly seen as offering a viable alternative treatment strategy with on-going clinical trials to treat disorders such as inflammatory bowel disease (IBD).

Successful encapsulation of *E. coli* T3 phages reported here using L-55, L100 and S100 polymers opens-up the possibility for more precise targeted delivery of therapeutic phages. Encapsulation of phage payloads ~10^8^ PFU/g with controlled release triggered due to differences in the gut pH was shown; L-55 released at pH 5.5 and above, with L100 releasing at pH 6 and above and S100 capsules capable of delivering phages further down the GI tract, e.g., large intestine ~pH 7. The stratified release characteristics would allow more precise control over phage payload delivery in the GI tract. Capsules made from L-55 and L100 may be suitable for in vivo studies involving small animal models, e.g., mice and rats; the intestinal physiological pH values in these animals is well suited to these polymer dissolution profiles. L-55 and L100 capsules would also allow delivery of therapeutic cargo to the different parts of the small intestine in humans ((proximal pH 5.8–6.5); distal (pH 6.6–7.5)) [28]. S100 capsules provide the possibility of delivering payloads further down the GI tract in the large intestine where the pH is ~7, allowing site specific delivery strategies to be considered. Phage release kinetics confirmed complete release within 30–45 min of exposure of the capsules at the relevant pH conditions indicating burst release. The time lapse microscope images showed immediate release from individual S100 capsules upon exposure to pH 7.5. Observed agglomeration of capsules may slow the release dynamics, due to variations in the local pH values. This may result in phage distribution along the sections of the GI tract which in any case may be a desirable outcome [29].

The temperature and time of TSA induced protonation impacted on the yield of active phages and the acid protection characteristics of the capsules. Protonation was carried out in acidified oil and resulted in precipitation of the synthetic polymer. Increasing the curing temperature allowed faster protonation and resulted in capsules that afforded better phage protection upon exposure to SGF. Protonation time was found to be a critical parameter affecting final phage recovery yields and microcapsule gastric stability. Optimisation of the gelation (capsule curing) time and temperature resulted in high phage titres (10^7–8^ PFU/mL) in the final solid capsules after acid exposure. Crosslinking with CaCl_2_ was needed to induce alginate gelation [30]. Alginate crosslinking was a critical step in ensuring that the capsules afforded acid protection to encapsulated phages. Alginate crosslinking time of 1 h was found to be sufficient for phage protection from acid exposure and reduced the tendency of the capsules to aggregate. Reducing alginate crosslinking times was found to adversely impact on acid stability of the encapsulated phages. Alginate is commonly employed as a biopolymer for protection of biological therapeutics from environmental stresses and it’s naturally occurring pH responsiveness. Alginate aids gastric stability through shrinkage at low pH, which in turn protects the encapsulated phage, and exchange of crosslinking divalent ions (Ca^2+^) with monovalent cations (such as sodium ions) in alkaline pH cause structural disintegration [30] (Figure S4). Capsules prepared using the different polymer chemistries offered varying degrees of acid protection to the phages. Furthermore, alginate addition to the formulation was needed in addition to the synthetic polymers for acid protection. Capsules prepared without the addition of alginate gave insufficient acid protection to the encapsulated phage. S100 capsules offered the highest level of protection which may be attributed to lower amounts of carboxylic acid groups in the polymer backbone allowing a denser alginate-polymer network reducing proton permeability within the capsules. Exposure of the capsules to SGF (pH 1.5 and pH 2 for 2 h) showed significant acid protection for capsules prepared using all three polymers and suggests that high encapsulated phage pay loads could be delivered in vivo. Exposure of the capsules to acid resulted in changes in phage release kinetics suggesting further protonation of the polymer network accounting for the delayed phage release. Unencapsulated *E.coli* T3 phages exposed to pH 1.5 were not detectable after 5 min of acid exposure, the process of encapsulation had a marked effect on protecting the phages from acid damage.

There were only slight changes in encapsulated phage activity during storage at 4–8 °C for 3 months of testing, however, capsules tended to adhere and aggregate during storage which could be attributed to the presence of alginate which makes the capsules sticky. Therapeutics encapsulated in small microcapsules are particularly suitable for testing their in vivo efficacy in small animal models (e.g., rats and mice) and these tests necessitate using small diameter oral gavage tubes (e.g., gauge 18, internal diameter of needle bore 0.864 mm). Aggregation of microcapsules makes delivery using these oral gavage tubes challenging. Capsules stored at room temperature agglomerated significantly over a 2-week period of testing compared with those stored in the fridge. Residual CaCl_2_ in capsules may continue to crosslink alginate polymer chains within the capsules resulting in fusion with capsules in close proximity upon contact. The agglomerated capsules formed after room temperature storage were too large for oral gavage administration. On the other hand, potentially slower rates of crosslinking for refrigerated capsules resulted in only modest aggregation of capsules over a four-week storage period. Use of non-ionic surfactants such as Tween 20 in the resuspension buffer reduced observed agglomeration and enabled successful dispersion of the phage-loaded microcapsules for oral administration. Future work should investigate factors affecting capsule aggregation during storage including strategies to harden the capsules preventing their fusion. Factors affecting the hardening of the alginate capsules need further exploration. These may include increasing the molarity of the calcium chloride during the alginate crosslinking step from 0.1 M to 1 M which may result in rapid gelation and the harder capsules may reduce microcapsule agglomeration. The temperature of gelation may also be an important factor worthy of further investigation.

## 4. Materials and Methods

### 4.1. Escherichia coli and Bacteriophage T3 Propagation

*Escherichia coli* strain ATCC11303 and its lytic phage T3 (ATCC11303-B3, family Podoviridae) were purchased from LGC Standards (Teddington, Middlesex, UK). *E. coli* was streaked onto LB agar (25 g/L LB broth Miller, Fisher Scientific UK, Loughborough, UK) with 1.5 *w/v*% Bacteriological Agar No. 1 (Oxoid, Basingstoke, UK) and incubated overnight in the static incubator at 37 °C. For liquid cultures, one colony or 10 µL of a previous liquid culture was added to a sterile flask containing 25 mL of LB broth. This was incubated in a shaking incubator (Certomat^®^ BS-1, Sartorius, Surrey, UK) overnight at 37 °C 150 RPM. The following day, the overnight culture was centrifuged at 2000× *g* for 20 min and the supernatant was subsequently removed and resuspended in SM buffer (100 mM NaCl, 8 mM MgSO_4_, 50 mM Tris-HCl, pH adjusted to 7.5 using 4M HCl) for refrigerated storage.

Bacteriophage T3 was propagated through preparation of a fresh overnight culture of *E. coli.* Absorbance (OD_600nm_) measurements using a spectrophotometer (UV Mini 1240, Shimadzu, Milton Keynes, UK) were taken every 30 min until the optical density reached a value of 0.2 (corresponding to 10^7^ CFU/mL). At this point, phage T3 was added at a multiplicity of infection (MOI) value of 0.001. 5 min after infection, a working concentration of 25 mM of ammonium sulphate (Fisher Scientific, Loughborough UK) was added. The flask was placed back into the incubator and the absorbance measured every 30 min until the optical density decreased to approximately 0.05 and remained stationary. The phage culture was then transferred into a sterile centrifuge tube (50 mL Falcon, Fisher Scientific UK) and centrifuged 2000× *g* for 20 min. After this, the supernatant was filtered using a 0.22 µm membrane. Phage stocks were concentrated using a 100 kDa ultrafiltration Amicon tube (Fisher Scientific UK) and diafiltration completed with the addition of SM buffer (100 mM NaCl, 8 mM MgSO_4_, 50 mM Tris-HCl, pH 7.5) during 5 repeated centrifuge cycles of 2000× *g* for 20 min.

Final phage titres were confirmed using the double layer agar method [31]. In a sterile tube, typically 5ml of LB top agar (LB broth, Fisher Scientific UK with 0.5% Bacteriological Agar No. 1, Oxoid, Basingstoke, UK) was mixed with 5 mL salt solution (400 mM MgCl_2_ and 100 mM CaCl_2_). 10 µL of *E. coli* culture was added to this mixture and poured over a LB agar plate and dried under a laminar flow hood. The phage sample was serially diluted in a 96 well plate from dilution factor 10^−1^ to 10^−8^. 10 µL of each phage dilution was spotted in quadruplicate on the agar plate and incubated overnight at 30 °C or for 4 h at 37 °C.

### 4.2. Chemical Reagents

Miglyol 840, purchased from Safic Alcan UK (Warrington, Cheshire, UK), is a propylene glycol diester of saturated plant fatty acids, forming the continuous phase. Polyglycerol polyricinoleate (PGPR) an emulsifier produced from glycerol and fatty acids was purchased from Aston Chemicals (Aylesbury, Buckinghamshire, UK). Eudragit L 100–55 (L-55) contains an anionic copolymer based on ethyl acrylate and methacrylic acid, and was purchased from Evonik (Essen, Germany). Eudragit polymers L100 and S100 are methyl methacrylate co-methacrylic acid copolymers. Medium viscosity alginate was purchased from Sigma Aldrich (Gillingham, Dorset, UK). Sodium hydroxide, sodium chloride, hexane, tween 20, castor oil and p-Toluenesulfonic acid (pTSA) were all purchased from Fisher Scientific UK.

### 4.3. Production of Water-in-Oil Emulsion for the Encapsulation of T3 Phages Using the Process of Membrane Emulsification

The dispersed (aqueous) phase was composed of a Eudragit polymer and medium viscosity alginate. The polymer was initially dissolved in an alkaline solution at a concentration of 10% (*w/v*). This was typically produced in 40 mL batches with 36 mL dH_2_O, 4 mL of 4 M NaOH and 4 g polymer L-55 or L100. For production of S100, batches were made using 37 mL dH_2_O, 3 mL of 4 M NaOH and 4 g S100. This solution was mixed with a magnetic stirring bar until completely dissolved, observed by the solution changing from cloudy to clear. The pH was measured after polymer dissolution, usually reading approximately pH 6.5. Subsequently the alginate was added at a concentration of 1% (*w/v*) and mixed with a magnetic stirring bar overnight, usually by which the alginate had dissolved entirely. T3 at a PFU of approximately ~10^9^ PFU/mL was added to the dispersed phase at a ratio of 1:10 (*v/v*), respectively and mixed until fully dispersed. T3 phage was added immediately before the membrane emulsification process began to ensure minimum phage losses during production. The continuous (oil) phase was produced using miglyol and castor oil at a ratio of 9:1 (*v/v*), respectively. 5% PGPR (*w/v*) was added as an emulsifier, which lowered the interfacial tension between oil and water.

A membrane emulsification dispersion cell LDC-1 (Micropore Technologies Ltd., Redcar, UK) was utilised to produce a water-in-oil (W/O) emulsion (Figure 1). A stainless-steel membrane was selected with uniformly spaced 40 µm micropore arrays in a ringed formation. To begin with, the membrane was submerged in 1H, 1H, 2H, 2H-Perfluorodecyltriethoxysilane (Sigma Aldrich, Dorset, UK) to produce a hydrophobic surface. This membrane coating ensured droplets would detach from the membrane, into the continuous phase as opposed to spreading across the membrane surface. A syringe containing the dispersed phase was used to fill the cavity beneath the membrane, eliminating the presence of air bubbles. 50 mL of the continuous phase solution was pipetted into the cylindrical glass chamber above the membrane. A paddle blade created shear across the membrane using controlled rotation speed of 250 RPM. 5 mL of the dispersed phase was then driven upwards through the 40 µm pores using a syringe pump (Harvard Apparatus, Cambridge UK) at a flow rate of 25 mL/h to form a water-in-oil (W/O) emulsion. The method of laser diffraction was selected to measure emulsion characteristics, using the Beckmann LS coulter machinery. Glass beads were used as control samples to confirm the reliability and accuracy of the method.

### 4.4. Phage Encapsulation and Capsule Production Process

After the initial emulsion production, a sample of each emulsion was taken for capsule size analysis. Protonation of the polymer then took place using 0.05 M pTSA, Miglyol and 5% (*w/v*) PGPR in an excess volume (Figure 1b). This process caused polymer precipitation by which the pH-responsive microcapsules are produced. The emulsion mixed with 0.05 M pTSA was stirred at 100 RPM using axial mixing for 2 h at a controlled temperature of 37 °C.

Hexane was added 50:50 (*v/v*) in a beaker with the protonated solution in which the microcapsules formed a precipitate and remained at the base of the beaker (Figure 1c). Hexane was then carefully aspirated, leaving a capsule precipitate, which was resuspended using 2% (*v/v*) tween 20 (pH 4). To minimise the possibility of particle aggregation during this processing stage, the sample was gently mixed at 100 RPM using a magnetic stirrer throughout resuspension to produce a dispersed sample. A final working concentration of 0.1 M CaCl_2_ was added to the solution for alginate crosslinking (Figure 1c). This solution was then mixed using the same three-bladed impeller at 100 rpm for 1 h. The final capsules were then washed 3 times volume using the 2% tween solution (pH 4) to remove residual CaCl_2_. Finally, the capsules in 2% tween were transferred into falcon tubes in which the capsules settled at the base of the tube. Any liquid was removed carefully with a pipette and the capsules were stored in the refrigerator.

### 4.5. Particle Size Analysis of Encapsulated Phage Containing Capsules

During the microcapsule production process, samples were taken to observe and analyse using a high-speed camera (Micro C100 Phantom Ametek, UK) which was connected to a Nikon Eclipse E200 microscope. The x 10 magnification lens was used to view the samples and images were captured through connection to a laptop and use of the Phantom Camera Control software (PCC 3.1). The size distributions of droplets and capsules were measured through laser diffraction using the Coulter LS series 130 (Beckmann Coulter Inc., High Wycombe, UK). 15 mL of miglyol + 5% (*w/v*) PGPR was placed into the coulter vessel and the offsets and background were measured. The emulsion sample was added until the obscuration measured between 8–12% and the droplet size distributions were then generated. For capsule size characterisation, 15 mL of 2% (*v/v*) tween solution (pH 4) was used to set the background and suspended capsules were added until the correct obscuration was achieved. Each distribution was measured three times and averaged to produce final size distribution curves. Statistical analysis of the distributions was completed in Excel to conclude mean diameters, coefficient variants and standard deviations.

### 4.6. Release Kinetics of Encapsulated T3 upon Exposure to Simulated Intestinal Fluid

The phages were released from the microcapsules by exposure to Sorensen’s buffer (0.2 M sodium phosphate and 0.2 M sodium phosphate dibasic) at pH 5.5, 6 or 7, dependent upon the polymer used, for 2 h. Typically, 0.1 g of microcapsules were added to 1 mL of Sorensen’s buffer. Time points were taken every 10 min for 30 min and then every 15 min for the remaining duration. For each timepoint, the supernatant was diluted 10-fold using SM buffer from 10^−1^ to 10^−8^. The dilutions were plated in quadruplicate using the double layer agar method and incubated overnight at 30 °C or for 4 h at 37 °C.

### 4.7. Exposure of Capsules to Simulated Gastric Fluid to Evaluate Phage Survival Due to Acid Exposure

Typically, 0.1 g of T3 containing microcapsules were added to a sterile bijoux (7 mL, Fisher Scientific, Loughborough, UK) along with 1 mL of SGF (Sorensen’s buffer with pH adjusted using HCl) at pH 1.5 and 2. Buffers used for acid exposure were adjusted using 1 M HCl and the final pH was measured with a pH meter (pH 1.5 and 2). The bijoux containing hydrogel microcapsules and SGF was incubated at 37 °C in a shaking incubator (Certomat, BS-1, Sartorius, UK) for 2 h. The capsules were pelleted using centrifugation and the supernatant was removed. The pelleted capsules were resuspended in 1 mL of Sorensen’s buffer (pH 5.5, 6 or 7) and incubated further for 2 h at 37 °C. Phage titres were confirmed using the standard plaque double overlay assay (described previously).

### 4.8. Stability of Encapsulated T3 Phages Stored in the Fridge over Six Months

To investigate the storage stability of encapsulated T3 phages in each polymer, typically 3 batches of 1 g of microcapsules were placed in sealed 15 mL falcon tubes and stored at 4 °C for a period of 6 months. Samples were taken weekly for the first month then at 3 months and 6 months, dissolved in Sorensen’s buffer as indicated above and the final phage titre confirmed using plaque assays. The capsule size examinations were carried out using a LS150 coulter instrument (as outlined previously).

## 5. Conclusions

*E-coli* specific bacteriophage T3 was successfully encapsulated in three different pH-responsive polymers with added alginate biopolymer conferring improved acid stability. The critical process parameters for the membrane emulsification were optimised to facilitate high phage encapsulation yields. The selected pH-responsive polymer formulation resulted in phage release triggered at different solution pH values mimicking different sites of infection in the gastrointestinal tract of humans and animals. The capsules were found to be acid stable, enabling T3 phage survival at low acidic pH values as low as 1.5 mimicking gastric acidity conditions in humans and animals such as in poultry. Storage and agglomeration assays confirmed high levels of phage viability four weeks after production, with limited agglomeration over this storage period. Further work is needed to evaluate factors affecting the agglomeration of capsules as well as in vivo studies evaluating the delivery of phages, e.g., in small animal models.

## Figures and Tables

**Table 1:** Summary table of emulsion size statistics.

Polymer	L-55	L100	S100
Mean (µm)	96	86	101
D-10 (µm)	67	57	53
D-50 (µm)	102	90	113
D-90 (µm)	132	113	166
Span (D90-D10/D50)	0.64	0.62	1.0
Standard Deviation (µm)	26	26	39
Coefficient of variation(%)	27	30	39

## Data Availability

All relevant data are included in the manuscript. Raw data can be made available upon request from the corresponding author.

## Supplementary Materials

The following are available online at https://www.mdpi.com/article/10.3390/ph14050424/s1, Figure S1: Final capsule phage recovery with and without alginate. Figure S2: SEM images of freeze-dried S100 (top left and top right) and L100 capsules, Figure S3: chemical structures of Eudragit polymers S100, L100 and L-55., Figure S4: Chemical processes involved in capsule production and subsequent release., Table S1: Investigating protonation conditions to determine the optimal parameters for high phage yields after production of capsules and stability upon exposure to SGF.
