# Supplementary material for: Microencapsulation of Bacteriophages Using Membrane Emulsification in Different pH-Triggered Controlled Release Formulations for Oral Administration
Source: Pharmaceuticals (Basel). 2021 May 2;14(5):424. doi: 10.3390/ph14050424 (PMC8147480; doi:10.3390/ph14050424)
Supplement: Supplementary file 1 [file pharmaceuticals-14-00424-s001.zip › pharmaceuticals-1172081-supplementary.pdf]

Supplementary data

Table S1. Investigating protonation conditions to determine the optimal parameters for high phage yields after production of capsules and stability upon exposure to SGF. Capsules were composed of 10% (w/v) S100 with 1% (w/v) alginate and protonated using Miglyol + 5% PGPR + 0.05M pTSA. The pTSA induced protonation was carried out at a controlled temperature of either 25 ^{0}C or 37 ^{0}C, and pTSA exposure for 2, 4 and 6 h before alginate crosslinking and capsule recovery. The final capsules were dissolved in SIF (pH 7) to determine the phage titres in the capsules after production. Capsules were exposed to pH 1.5 or pH 2 SGF for 2 h in a shaking incubator at 37 ^{0}C before removal of acid and release of phages in SIF.

Temperature of protonation (^{0}C)	Time of protonation (h)	Phage titre in capsules after production (PFU/g)	Phage titre in capsules after exposure of capsules to pH 1.5 (PFU/g)	Phage titre in capsules after exposure of capsules to pH 2 (PFU/g)
25	2	2.3×10^8	0	0
25	4	1.3×10^8	4.5×10^4	6.0×10^4
25	6	4.3×10^7	8.0×10^6	9.3×10^6
37	2	8.3×10^8	6.3×10^8	5.9×10^8
37	4	1.2×10^8	4.8×10^7	4.1×10^7
37	6	8.0×10^4	1.6×10^4	3.0×10^4

(a)

Alginate concentration (w/v)	Initial capsule titre (PFU/g)
0%	0
1%	8.3×10^8

(b)

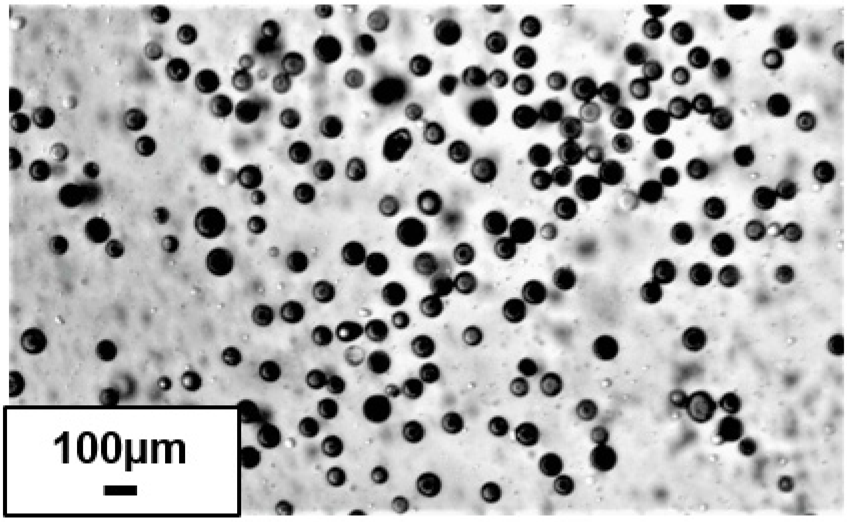

Figure S1. Final capsule phage recovery with and without alginate. (a) No phage could be detected after production without the addition of alginate to the formulation. 1% alginate offered minimal phage loss during production. 2% alginate resulted in membrane fouling. (b) Optical image of capsules produced with 10% (w/v) S100 and no alginate in the formulation.

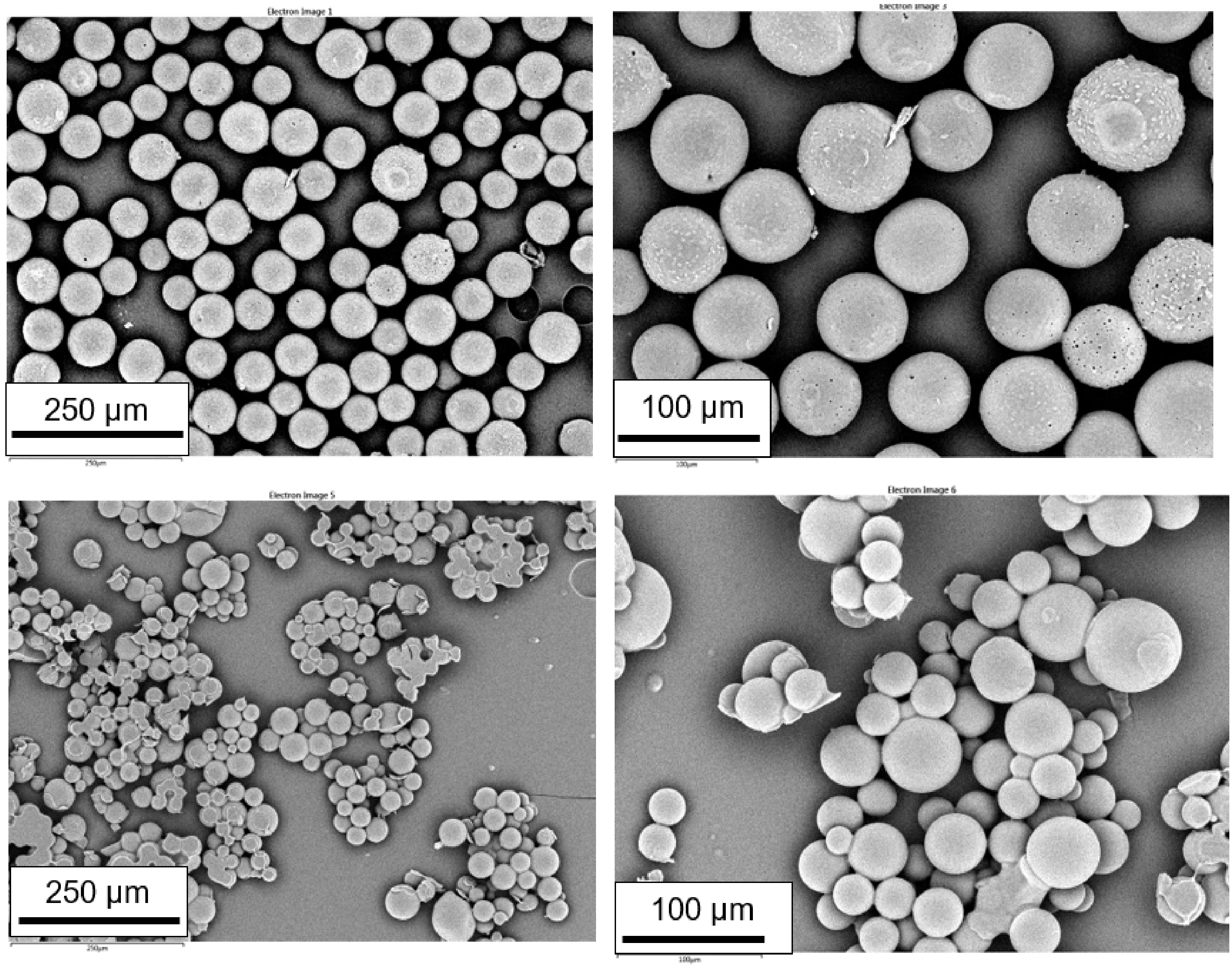

Figure S2. SEM images of freeze-dried S100 (top left and top right) and L100 capsules (bottom left and bottom right) to examine the agglomeration of capsules composed of different polymers. Capsules were produced using 10% (w/v) S100 or L100 with 1% (w/v) alginate before freeze drying for 24 h. The capsules were collected and examined using SEM analysis to visualise the agglomeration.

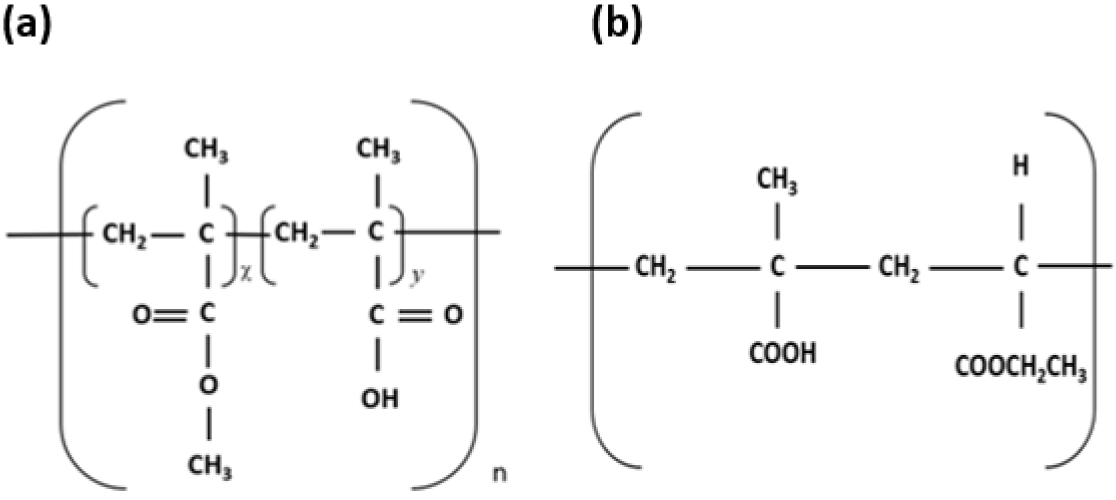

Figure S3. Chemical structure of Eudragit polymers. (a) L100 and S100 co-polymers with differing ratios of free carboxyl (y) groups to ester (x) groups (L100 ratio 1:1 and S100 ratio 1:2). (b) L-55 chemical structure. 'n' represent number of sub units.

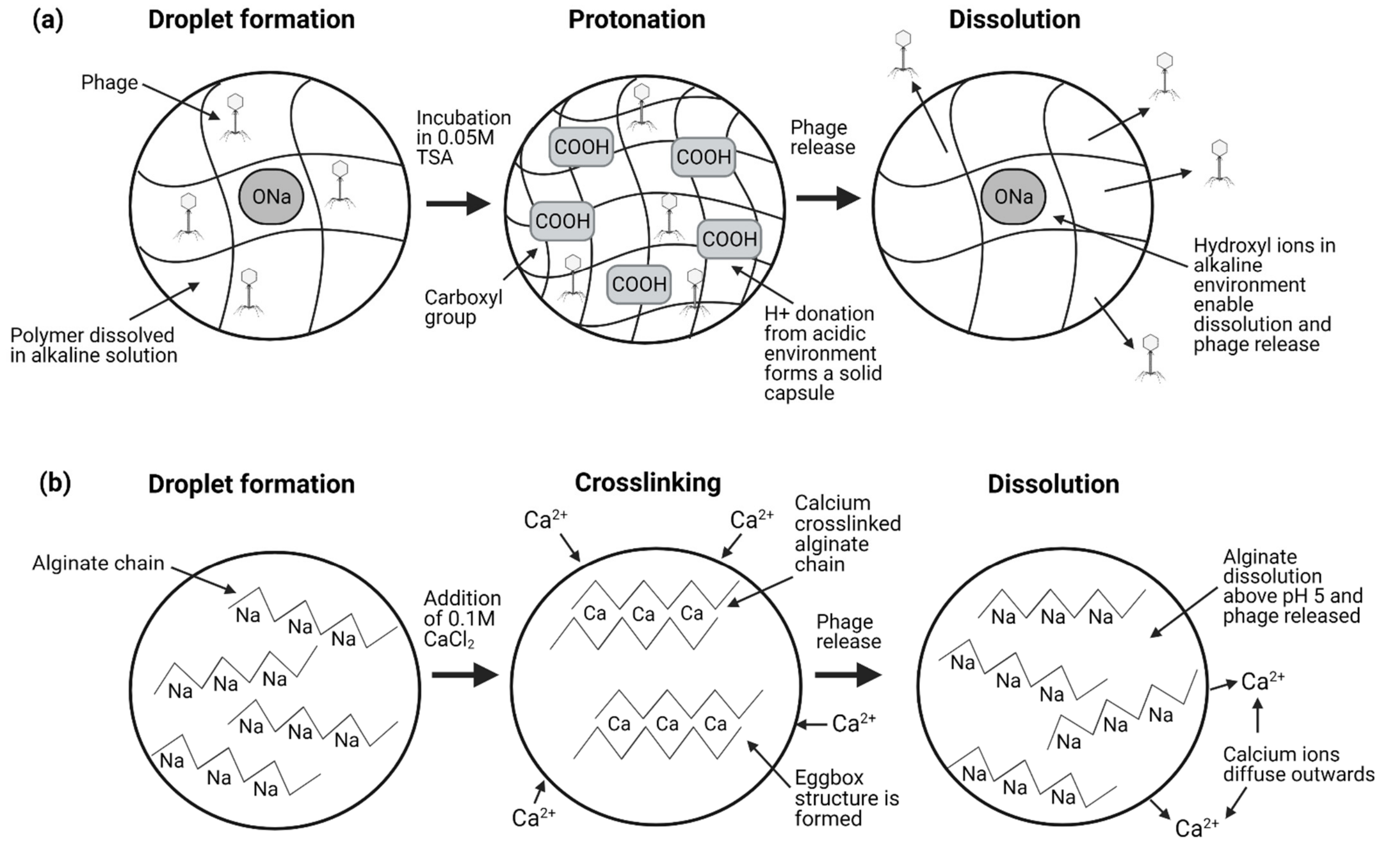

Figure S4. Chemical processes involved in capsule production and subsequent release due to pH triggered dissolution. (a) Eudragit polymer protonation and dissolution, (b) alginate crosslinking and dissolution. Created with BioRender.com
